# ‘Next-generation’ genome wide association studies

**DOI:** 10.1186/1897-4287-10-S2-A48

**Published:** 2012-04-12

**Authors:** J Hopper, E Makalic, D Schmidt, M Bui, J Stone, M Kapuscinski, D Park, M Jenkins, M Southey

**Affiliations:** 1 The University of Melbourne, Departments of Public Health and Pathology, Australia

## 

The first wave of cancer genome-wide association studies (GWAS) have revealed tens of independent loci marked by common variants of unknown or likely no functional significance that explain about 5-10% of familial risk for the particular disease. The approach taken to date has been conservative, and only a fraction of information has yet to be extracted from these expensive enterprises. For example, the Bonferroni procedure for selecting candidate phase II SNPs ignores many SNPs that happen to fail an extremely low *p*-value threshold. While this procedure does guarantee control of false positives, it seems counterintuitive to the purpose of phase I, which is to generate hypotheses based on promising candidates. Researchers have generally combined data from the discovery phase I and other phases and used ‘genome-wide thresholds’ based on assuming all SNPs are independent. Linkage disequilibrium (LD) makes it problematic to differentiate a real signal from highly correlated proxy signals. Most published GWAS do not examine SNP interactions due to: (a) the high computational complexity of computing *p*-values for the interaction terms, and (b) the typically low power to detect significant interactions. It is plausible that more information should be extracted if: (i) higher order interactions are fitted, (ii) highly selected cases and controls are used in phase I, (iii) large replication studies are used, especially if involving existing GWAS data, (iv) the non-independence of SNPs is taken into account using, e.g. BEAGLE CALL or haplotype analyses, (v) focus is on candidate gene pathways, and/or functional SNPs, and (vi) rarer and more SNPs, such as is available from the Illumina 5M SNP chip, are used. We will illustrate these ideas using data from a GWAS of early-onset breast cancers, enriched for those with a family history, and a GWAS using extremes sample of extremes for mammographic density. We will also discuss the design of a large international breast cancer GWAS using the Illumina 5M SNP chip, phase I cases enriched for family history, population-based phase II cases and controls, population-based family study of candidate SNPs, and GxG analyses using ‘massively parallel’ super computing.

